# Cerebral Perfusion Pressure in Severe Traumatic Brain Injury Survivors and Non-Survivors: A Meta-Analysis

**DOI:** 10.3390/brainsci15111161

**Published:** 2025-10-29

**Authors:** Maria Karagianni, Alexandros G. Brotis, Charikleia S. Vrettou, Kerasia Goupou, George Stranjalis, Kostas N. Fountas

**Affiliations:** 1 Department of Neurosurgery, General University Hospital of Larissa, 41110 Larissa, Greece; 2 Medical School of Thessaly, University of Thessaly, 41110 Larissa, Greece; 3 First Department of Critical Care Medicine and Pulmonary Services, Evangelismos Hospital, Medical School of National and Kapodistrian University of Athens, 45–47 Ipsilantou St., 10676 Athens, Greece; 4 Faculty of Medicine, School of Health Sciences, University of Thessaly, Biopolis, 41110 Larissa, Greece; 5 Athens Microneurosurgery Laboratory, Evangelismos Hospital, 10675 Athens, Greece; 6 Department of Neurosurgery, Evangelismos Hospital, National and Kapodistrian University of Athens, 10676 Athens, Greece; 7 Department of Anatomy, Medical School, National and Kapodistrian University of Athens, 11527 Athens, Greece; 8 Hellenic Center for Neurosurgical Research, “Petros Kokkalis”, Evangelismos Hospital, 10675 Athens, Greece

**Keywords:** severe traumatic brain injury (sTBI), cerebral perfusion pressure (CPP), Glasgow Outcome Scale (GOS), meta-analysis, neurocritical care, autoregulation, patient outcome

## Abstract

Background: Severe traumatic brain injury (sTBI) is a leading cause of death and disability worldwide. Cerebral perfusion pressure (CPP), the difference between mean arterial and intracranial pressure, is crucial for maintaining cerebral blood flow. However, the optimal CPP threshold for improving outcomes remains uncertain. Objective: To identify CPP levels associated with favorable outcomes following sTBI through a systematic review and meta-analysis. Methods: Following PRISMA guidelines, we systematically searched PubMed, Scopus, and Web of Science up to February 2024 for studies involving adult sTBI patients admitted to intensive care units. Studies reporting CPP in relation to outcomes measured by the Glasgow Outcome Scale (GOS) were included. Pooled mean CPP differences between outcome groups were calculated using a random-effects model. Study quality was assessed using the Newcastle–Ottawa Scale, and evidence certainty was evaluated with GRADE. Results: Twenty-two studies with 2986 patients met inclusion criteria. Patients with good outcomes (GOS > 3) had higher CPP (77.5 mmHg; 95% CI: 73.8–81.2) than those with poor outcomes (67.2 mmHg; 95% CI: 60.4–74.1), with a mean difference of 10.01 mmHg (95% CI: 4.23–15.80; *p* < 0.05). Survivors also demonstrated higher CPP than non-survivors (mean difference 8.15 mmHg; 95% CI: 3.28–13.02). Evidence quality ranged from low to very low due to study heterogeneity. Conclusions: Higher CPP levels (~75–80 mmHg) are associated with better survival and functional outcomes after sTBI, supporting individualized, multimodal CPP management rather than a fixed 60 mmHg threshold.

## 1. Introduction

Severe traumatic brain injury (sTBI) is a major global health concern, frequently resulting in substantial morbidity and mortality [1]. It is characterized by extensive brain tissue damage from significant external forces and presents a complex clinical challenge for healthcare professionals [2]. Beyond the primary injury, secondary insults—such as hypoxia and hypotension—can exacerbate neurological damage and worsen prognosis, highlighting the need for timely and targeted interventions [3].

A key physiological parameter in the management of sTBI is cerebral perfusion pressure (CPP), defined as the difference between mean arterial pressure (MAP) and intracranial pressure (ICP) [4]. CPP represents the driving force for cerebral blood flow and is essential for maintaining adequate oxygen and nutrient delivery to the injured brain [5,6]. Historically, maintaining CPP above 60 mmHg has been the standard therapeutic target [7]; however, emerging evidence indicates that the optimal CPP threshold may vary between patients. Both insufficient CPP, which risks cerebral ischemia, and excessive CPP, which can contribute to cerebral edema and acute respiratory distress syndrome, have been associated with poor neurological outcomes [6,8,9].

While adjunctive modalities such as brain tissue oxygen monitoring, cerebral microdialysis, and advanced neuroimaging are increasingly employed to refine treatment, the precise CPP levels associated with the best clinical outcomes remain a matter of ongoing debate [6,8,9]. Understanding the relationship between CPP and patient prognosis is therefore critical for guiding individualized therapy and improving survival and recovery.

In this context, our meta-analysis aims to synthesize existing evidence to determine CPP thresholds associated with improved outcomes following sTBI. Specifically, we compare CPP values during ICU stay between patients with good outcomes (GOS > 3) and those with poor outcomes (GOS 3–1), between patients with excellent outcomes (GOS 5) and non-survivors (GOS 1), between patients with favorable outcomes (GOS 4–5) and those with GOS 1, and between survivors (GOS 5–2) and non-survivors (GOS 1). By pooling data from diverse studies, this study seeks to provide clinicians with robust, evidence-based guidance for CPP management, ultimately aiming to enhance both survival rates and functional recovery in individuals with sTBI.

## 2. Materials and Methods

We conducted a meta-analysis using an electronic literature search that adhered to the PRISMA checklist [10]. The reporting of this systematic review and meta-analysis follows the PRISMA 2020 guidelines [10]. A completed PRISMA 2020 checklist is provided as Supplementary Table S1. The study was registered in Prospero, under the identification number: CRD42023391260. This study is independent and not based on any previous meta-analysis. Our systematic review’s approach to identifying studies is transparently reported in the manuscript.

### 2.1. Search Strategy—Information Sources

For the current meta-analysis, we focused on three main concepts: “severe TBI”, “CPP”, and “outcome”. To identify relevant articles, we searched Medical Subjects Headings/National Library of Medicine, Google Scholar, and Wikipedia for relevant keywords. Both MK and AB conducted independent searches of electronic databases (PubMed, Scopus, and Web of Science) using every possible combination of keywords. The search strings for each database are presented in Table 1. To ensure reproducibility, the full Boolean search strings for PubMed, Scopus, and Web of Science are reported exactly as used, without session-dependent or temporary links in Table 1. For completeness, Google Scholar was also searched using the same keywords as in the PubMed query (“cerebral perfusion pressure” AND “traumatic brain injury”), but this step was performed as a complementary and exploratory search. We also searched the reference lists of the collected articles to find additional relevant studies. We conducted a comprehensive literature search on 6 January 2024, and then reran the search on 22 February 2024.

### 2.2. Eligibility Criteria

We searched for randomized controlled trials (RCTs) or observational studies in English that focused on adults aged 18 and over with severe sTBI. Clinically, patients with sTBI are characterized by a Glasgow Coma Scale (GCS) score of 8 or less after initial resuscitation or upon admission. These patients were admitted to the intensive care unit, and the studies reported CPP measurement in both comparators. Our primary interest was the clinical endpoint of the Glasgow Outcome Scale (GOS) up to six months after the injury. Although RCTs were eligible, our search did not identify any RCTs meeting all inclusion criteria. Consequently, all included studies were observational in design. We only included human studies in English without any search filters.

The decision to restrict inclusion to English-language publications was based on pragmatic and methodological considerations. The review team’s fluency is limited to English and Greek, and no translation resources were available for accurate interpretation of studies published in other languages. Furthermore, most high-quality research in neurocritical care and traumatic brain injury is published in English, ensuring that our review captured the vast majority of relevant and high-impact evidence.

### 2.3. Selection Process

We uploaded the meta-data from all three databases in Rayyan [11]. Initially, we removed any duplicate studies across the three databases. In cases where multiple studies were based on the same study population, we retained only the latest article. Next, MK and AB screened the articles based on their title and abstract relevance. After that, MK and AB excluded studies based on their study design, such as laboratory studies, case series and reports, reviews, editorials, letters to the editor, and population, such as pediatric, non-traumatic, mild or moderate TBI. Similarly, they excluded studies that had no extractable data. Any disagreement between the two review authors was discussed with the senior author (KF).

### 2.4. Data Collection Process

The studies were identified by the name of the first authors and the year of publication. Two review authors, MK and AB independently collected the following data from each eligible study: the study design, sample baseline characteristics and social demographics, intervention details, cerebral perfusion measurements (mean values and standard deviation), patient outcome in GOS, and length of follow-up.

### 2.5. Evidence Synthesis

For every research question, we performed a proportion meta-analysis for each comparator and a comparative meta-analysis for the pooled estimate for continuous data. The outcome measure used was the CPP mean difference, and the results were presented through forest plots. We used the I^2^ statistic to measure the degree of statistical heterogeneity. We conducted rank correlation and regression tests to check for funnel plot asymmetry, using the observed outcomes’ standard error as predictors. Because of the variability among studies, we used a random-effects model and reported τ^2^ and 95% prediction intervals. Subgroup analyses were performed where possible, but meta-regression was not feasible due to limited and inconsistent data. We therefore focused on reporting random-effects estimates and confidence intervals to address heterogeneity transparently. The pooled estimates were reported along with their 95% confidence interval. All statistical analyses were conducted using the R statistical environment [12].

### 2.6. Risk of Bias Assessment

Two review authors (MK and AB) evaluated the risk of reporting bias in our study using two tools: the risk of bias tool for RCTs and the Newcastle Ottawa scale (NOS) for observational studies [13]. The NOS assesses three quality parameters (selection, comparability, and outcome) divided across eight specific items, which have slight differences in scoring case–control and longitudinal studies. Each item on the scale is scored from one point, except for comparability, which can score up to two points based on the specific topic of interest. Therefore, the maximum score for each study is 9, and studies with less than 5 points are considered high-risk for bias. Although tools like ROBINS-I offer more detailed assessments, we used the Newcastle–Ottawa Scale (NOS) as it remains a practical and widely accepted method for observational studies, especially when older data are inconsistently reported. A score of seven indicates low risk of bias, and these results informed the GRADE assessment without further downgrading, although the overall certainty of evidence remained very low. After consulting the senior author, we recorded the results in an Excel spreadsheet and resolved any discrepancies (Table 2). Because no eligible RCTs were identified, RCT-specific risk-of-bias tools were not applied. Finally, according to the GRADE recommendations, we assessed the overall risk of the evidence in our synthesized evidence (Table 3).

## 3. Results

### 3.1. Literature Search

**Figure 1:**
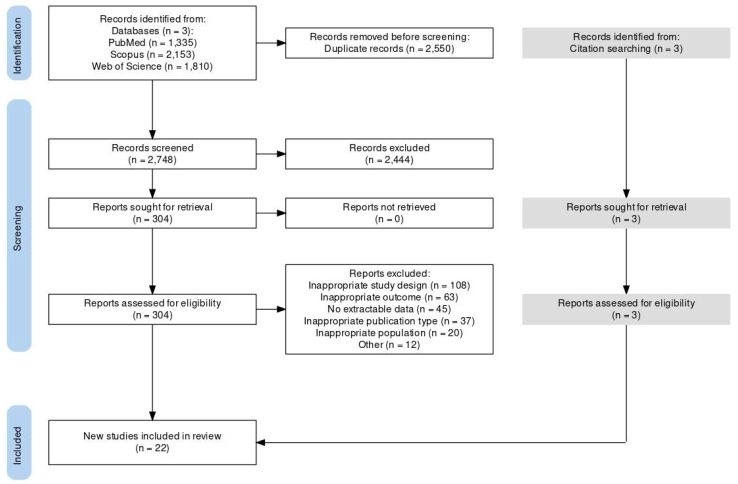
PRISMA 2020 flow diagram of literature search and study selection.

No randomized controlled trials met the eligibility criteria; all 22 included studies were observational. We conducted a literature search and found 2748 studies. After reviewing the title and abstract, we excluded 2444 articles. We read the full text of the remaining studies and excluded 285 more. We found three additional studies through references. Ultimately, 22 studies met our eligibility criteria and were included in our study. Figure 1 provides a visual representation of our screening process and Table 4 the characteristics of the eligible studies. 

### 3.2. Eligible Studies

All studies were written between 2002 and 2022 and followed a case–control study design, including 2986 patients who had suffered a sTBI and had undergone invasive neuromonitoring. Each study had a median of 56 patients, ranging from 15 to 556. The majority of studies (*n* = 7) were conducted in the UK, followed by the USA (*n* = 3), China (*n* = 2), and Lithuania (*n* = 2). Most studies used the Glasgow Outcome Coma Scale (GOS) to measure outcomes at the 6-month follow-up. According to the NOS, the eligible studies scored as high as seven (7), indicating a moderate risk of reporting bias.

### 3.3. Pooled Estimates

**Figure 2:**
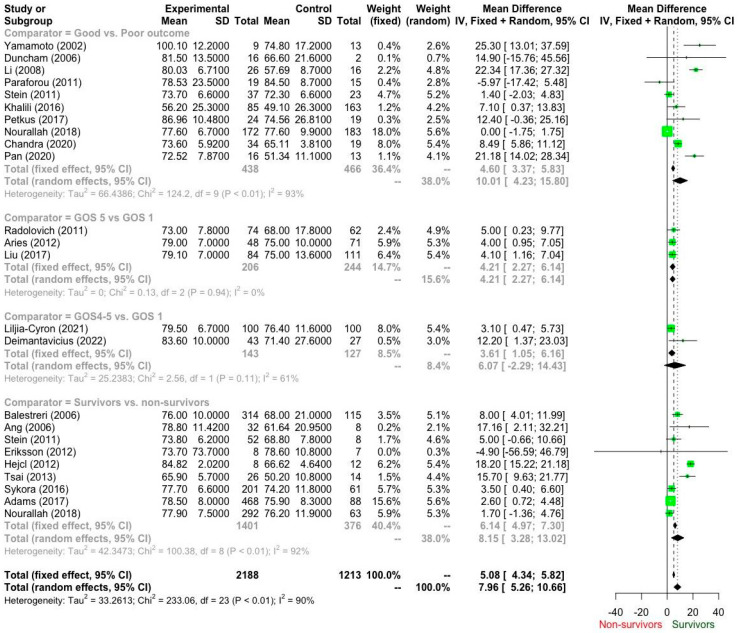
Forest plot of cerebral perfusion pressure (CPP) differences between patient outcome groups based on the Glasgow Outcome Score (GOS) [14,15,16,17,18,19,20,21,22,23,24,25,26,27,28,29,30,31,32,33,34,35].

According to the GRADE recommendations (Table 3), the available evidence ranges from “low” to “very low”. Ten studies (Table 4; Figure 2) addressed our primary question (Q1). Based on “very-low” quality of evidence, patients who had a good outcome required a CPP of 77.5 mmHg (95% CI: 73.78–81.2 mmHg) when compared to patients with poor outcomes who achieved a CPP of 67.2 mmHg (95% CI: 60.4–74.1 mmHg). The mean difference reached 10.01 mmHg (95% CI 4.23–15.80 mmHg; I^2^ 93%) and was statistically significant (*p* < 0.05).

### 3.4. Sensitivity Analysis

#### 3.4.1. Survivors vs. Non-Survivors: Subgroup Analysis by Country/Region

A subgroup analysis was conducted using a random-effects model to investigate if the country or region of the study influenced the overall effect size. The analysis included six distinct groups, comprising a total of nine studies (k = 9). The mean effect sizes for each subgroup were estimated using a random-effects model. tau^2^ and tau (the estimated variance and standard deviation of true effects) are reported for groups with more than one study (k > 1).

The analysis revealed notable variation in the mean effect sizes across the geographical groups (Table 5). Czechia showed the highest mean effect (Mean = 84.82), followed closely by Singapore (Mean = 78.80), while Taiwan exhibited the lowest mean effect (Mean = 65.90). For the subgroups with multiple studies (k > 1), estimates of true heterogeneity were calculated. The UK subgroup (k = 3) displayed evidence of low-to-moderate true heterogeneity, with an estimated true variance of tau^2^ = 1.20 (and tau = 1.09). The USA subgroup (k = 2) showed no true heterogeneity (tau^2^ = 0) in the estimated effects, suggesting the two included studies from this country were highly consistent. The remaining subgroups, which consisted of only one study, precluded the calculation of within-group heterogeneity (tau^2^ and tau). The formal test for subgroup differences was conducted to assess if the observed variation between country means was statistically significant. The between-groups heterogeneity test showed a substantial and highly statistically significant difference, confirming that the country or region of the study acts as a significant moderator of the effect size (Q = 231.71, df = 5, *p* < 0.0001). This finding strongly indicates that the magnitude of the measured effect varies significantly across the different geographical locations of the studies.

#### 3.4.2. Poor vs. Good Outcome: Subgroup Analysis by Country

A subgroup analysis was conducted to explore the influence of the study’s country of origin on the overall effect size. Initial testing for heterogeneity between the country subgroups using a fixed-effect model indicated a highly statistically significant difference in the mean effect across groups (Q = 130.13, df = 7, *p* < 0.0001). Significant heterogeneity was also present within the subgroups in this model (Q = 14.91, df = 2, *p* = 0.0006). To account for potential within-subgroup heterogeneity, mean effect sizes for each country were estimated using a random-effects model, where k is the number of studies in the subgroup.

The analysis revealed considerable variation in the mean effect sizes across the countries (Table 6). Japan exhibited the highest mean effect (Mean = 100.10), while Iran showed the lowest (Mean = 56.20). For the subgroups containing more than one study (USA and China), estimates of within-group heterogeneity were calculated. Both the USA (tau^2^ = 24.14, tau = 4.91) and China (tau^2^ = 25.40, tau = 5.04) showed moderate true variance in effect sizes (tau^2^) within their respective country groups. The remaining countries consisted of only one study (k = 1), thus precluding the calculation of within-group heterogeneity (tau^2^ and tau). The formal test for subgroup differences using the random-effects model confirmed that the country of origin was a highly significant moderator of the effect size. The between-groups heterogeneity test showed a substantial and statistically significant difference (Q = 124.09, df = 7, *p* < 0.0001). This result strongly supports the conclusion that the effect size varies significantly depending on the country in which the study was conducted.

Moreover, three studies compared patients with excellent outcomes (GOS 5, CPP 77.0 mmHg; 95% CI 73.1–81.0 mmHg) and patients with GOS 1 (CPP 73.17 mmHg; 95% CI 69.69–76.65 mmHg) (Q2). The mean difference was 4.21 mmHg (95% CI, 2.27–6.14 mmHg) in favour of patients with excellent outcomes. Notably, these results were based on “low” quality evidence.

Likewise, two studies addressed Q3. Based on “low” quality evidence, patients with GOS 4–5 (CPP 81. 3 mmHg; 95% CI 77.3–85.3 mmHg) recorded higher (mean difference 6.07 mmHg; 95% CI −2.29–14.43 mHg; I^2^ 62%) than patients with GOS 1 (CPP 76.17 mmHg; 95% CI 73.95–78.39 mmHg). However, this difference did not reach statistical significance (*p* = 0.155).

Finally, nine studies compared survivors to non-survivors (Q4), provided a “low” quality of evidence that survivors (76.7 mmHg; 95% CI 74.2–79.2 mmHg) required a higher CPP (8.15 mmHg; 95% CI 3.28–13.02 mmHg; I^2^ 92%) than non-survivors (69.42 mmHg; 95% CI; 64.88–73.97 mmHg).

**Figure 3:**
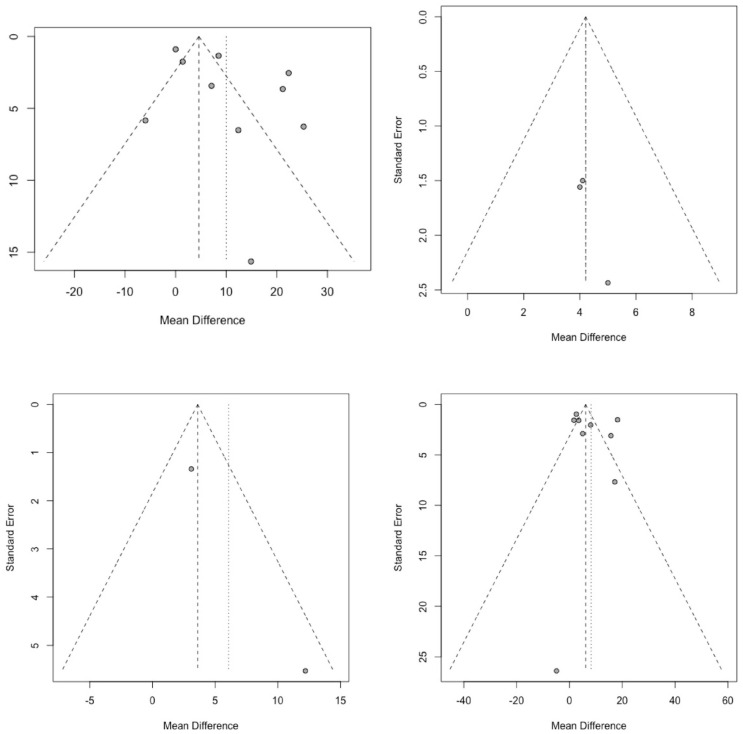
Funnel plots assessing publication bias for cerebral perfusion pressure (CPP) outcome comparisons. (Each dot in the funnel plots represents an individual study’s effect size plotted against its precision, with the central dashed line showing the pooled mean effect and the diagonal lines marking the 95% confidence limits).

After examining the funnel plots, publication bias could not be ruled out. However, Begg’s test identified no publication bias regarding our primary question (Q1) [36]. In the other research questions, the number of eligible studies precludes the use of a quantitative analysis of publication bias (Figure 3).

## 4. Discussion

### 4.1. Overview of Our Findings

Our meta-analysis consists of 22 studies and 2986 patients and suggests that higher cerebral perfusion pressure (CPP) values are associated with better outcomes following severe traumatic brain injury (sTBI). For our primary question (Q1), addressed by ten studies, patients with favorable outcomes (GOS > 3) exhibited a statistically significant mean CPP of 77.5 mmHg, compared to 67.2 mmHg in those with poor outcomes, yielding a mean difference of 10.01 mmHg (*p* < 0.05). Similarly, survivors required a higher CPP than non-survivors, with a mean difference of 8.15 mmHg.

Three studies comparing excellent outcomes (GOS 5) to poor outcomes (GOS 1) revealed a mean CPP difference of 4.21 mmHg, favoring the former. Two studies comparing GOS 4–5 and GOS 1 demonstrated a non-significant CPP difference. These results challenge the conventional practice of maintaining CPP above 60 mmHg, indicating that such levels may be insufficient for optimal neurological recovery. On the contrary, our analysis suggests that CPP values of 75 mmHg and above are associated with better outcomes in sTBI.

While the quality of evidence for the primary analysis was graded as “very low,” the consistency of findings across multiple subgroup comparisons underscores the potential benefit of adopting more aggressive CPP targets. Our results also indicate that survivors tend to have higher CPP values than non-survivors, though the evidence quality for this observation was “low”.

### 4.2. Comparison with the Literature

The current Brain Trauma Foundation guidelines recommend maintaining CPP between 60 and 70 mmHg [5], while the American Association of Neurological Surgeons proposed a minimum target of 60 mmHg [37]. However, the recent literature emphasizes the concept of an “optimal CPP” (CPPopt), determined by continuous autoregulation monitoring, as a more personalized approach to CPP management in sTBI [22,29]. Our findings agree with other studies reporting that favorable outcomes occur when CPP is maintained above CPPopt thresholds, whereas increased mortality is associated with CPP values below CPPopt [34].

By searching the literature, we have found several studies that provide further context. Petkus et al. [28] reported that CPP values of 85–95 mmHg are associated with better outcomes, although CPP > 95 mmHg may not confer additional benefit. Likewise, Ang et al. [16] observed that survivors consistently exhibited elevated CPP values compared to non-survivors, though extremely high CPP was associated with increased disability risk. Balestreri et al. [15] noted that excessive CPP may contribute to unfavorable outcomes, highlighting the need for individualized targets.

Our meta-analysis indicates that patients with better outcomes tended to maintain significantly higher CPP values (approximately 77.5 mmHg) compared with those with poor outcomes. Although cerebral autoregulation was not directly evaluated in our dataset, prior literature suggests that such higher CPP targets are most beneficial when autoregulation is preserved.

### 4.3. Implementation of Our Findings

These findings and their clinical implications are significant. Physicians managing sTBI may need to reconsider current CPP targets, because maintaining CPP between 60 and 70 mmHg—or a fixed goal of 60 mmHg—may be inadequate for optimal neurological recovery. In selected patients, especially those with preserved autoregulation, higher CPP targets may reduce mortality and improve functional outcomes [38]. Bögli et al., analyzing data from 809 adult TBI patients, found that patients are more at risk when their cerebral perfusion pressure (CPP) drops below personalized thresholds (CPPopt) than when it rises above them [39]. Based on this, the study recommends that individualized CPP management should focus primarily on preventing low CPP levels by using CPPopt as a lower safety limit [40].

However, aggressively increasing CPP carries the risk of complications such as cerebral edema or acute respiratory distress syndrome (ARDS), underscoring the importance of individualized treatment plans [41,42]. Continuous multimodal neuromonitoring—encompassing intracranial pressure (ICP), cerebral blood flow, brain tissue oxygenation, and autoregulation status—should guide CPP optimization strategies [43].

Personalized management algorithms incorporating CPPopt and the difference between actual CPP and CPPopt may further refine treatment, enabling clinicians to intervene when perfusion is suboptimal and to avoid potential harm from excessive CPP.

### 4.4. CPP and LMICs

Applying these findings in low- and middle-income countries (LMICs) presents distinct challenges. While our data suggest that targeting CPP near 77.5 mmHg may be beneficial, resource limitations often preclude advanced neuromonitoring. In such settings, a pragmatic approach involves maintaining CPP above 60 mmHg while closely monitoring clinical signs of cerebral hypoperfusion, such as pupillary changes and neurological deterioration [44].

Initiatives, including the training of healthcare providers in TBI management and protocol-based CPP monitoring, can help bridge resource gaps. Cost-effective measures—such as systematic use of available ICP monitoring tools, optimization of systemic oxygenation and blood pressure, and prevention of secondary brain injury—are especially relevant. By adapting evidence-based principles to local constraints, LMIC healthcare systems can improve survival and functional recovery after sTBI.

### 4.5. Gaps in the Literature

Despite these insights, substantial knowledge gaps remain. Few high-quality randomized controlled trials have directly compared different CPP targets, and most existing evidence is observational, limiting causal inference. The optimal CPP target likely varies by patient-specific factors such as age, injury severity, comorbidities, and autoregulation status—variables not consistently addressed in the available studies.

Long-term outcomes beyond six months remain poorly characterized, and the relationship between CPP and cerebral metabolism needs further exploration using advanced modalities such as brain tissue oxygen monitoring and cerebral microdialysis [44]. Future research should focus on prospective, multicenter trials to establish evidence-based CPP targets for specific patient subgroups and to assess the safety and feasibility of higher CPP thresholds in diverse healthcare settings.

To address the potential risk of cohort overlap—particularly among studies conducted in the United Kingdom—we performed a sensitivity analysis excluding studies with a high likelihood of overlapping cohorts. The results of this analysis were consistent with the main findings, supporting the robustness and reliability of our conclusions.

## 5. Conclusions

Several limitations must be considered when interpreting the results of this meta-analysis. First, the quality of evidence, as assessed by GRADE, ranged from “low” to “very low” across the included studies. This was due to factors such as the observational design of most studies, potential for selection bias, and heterogeneity in patient populations and treatment protocols. Second, while we conducted thorough searches, publication bias could not be eliminated, although Begg’s test did not identify significant publication bias for our primary question. The limited number of studies addressing secondary research questions precluded a quantitative analysis of publication bias for those outcomes. Third, most studies used the Glasgow Outcome Scale to measure outcomes at the 6-month follow-up, which may not fully capture the spectrum of long-term neurological and functional deficits following TBI. Moreover, no randomized controlled trials met our criteria, and the absence of experimental evidence limits causal inference. In addition, many studies lacked detailed reporting on how CPP was measured or averaged. Most did not specify whether values reflected daily means or overall ICU averages, or how multiple time points or management factors (such as decompressive craniectomy, sedation, or vasopressor use) were handled. As a result, we used the overall mean CPP reported in each study, which may have introduced additional heterogeneity. Finally, our analysis included studies conducted over a 20-year period (2002–2022), during which time there may have been changes in TBI management practices that could have influenced the observed CPP values and outcomes. These limitations underscore the need for cautious interpretation, as our findings represent observed associations rather than causal effects. Further research is needed to confirm and refine the relationship between CPP levels and clinical outcomes in TBI management.

## Figures and Tables

**Table 1:** Summary table of search string in the medical databases.

Database	Search String
Pubmed	(((“CPP”) OR (“cerebral perfusion pressure”)) OR (“neuromonitoring”)) AND ((((“cranial trauma”) OR (“head trauma”)) OR (“traumatic brain injury”)) OR (“TBI”))
Scopus	((TITLE-ABS-KEY (“cerebral perfusion pressure”) OR TITLE-ABS-KEY (“CPP”) OR TITLE-ABS-KEY (“neuromonitoring”))) AND ((TITLE-ABS-KEY (“head trauma”) OR TITLE-ABS-KEY (“cranial trauma”) OR TITLE-ABS-KEY (“traumatic brain injury”) OR TITLE-ABS-KEY (“TBI”)))
WoS	https://www.webofscience.com/wos/woscc/summary/2bd7d46a-331b-4e0c-9253-a6f0539535d3-c9e9b418/relevance/1 (accessed on 22 February 2024)

**Table 2:** Risk of bias assessment of included studies according to the Newcastle–Ottawa Scale (NOS).

Authors	Definition of Cases	Representativenes of Cases	Selection of Controls	Definition of Controls	Comparability	Ascertainment of Exposure	Same Method of Ascertainment in Cases and Controls	Non-Reposnse Rate	Total
Yamamoto (2002) [14]	1	1	1	1	0	1	1	1	7
Balestreri (2006) [15]	1	1	1	1	0	1	1	1	7
Ang (2006) [16]	1	1	1	1	0	1	1	1	7
Duncham (2006) [17]	1	1	1	1	0	1	1	1	7
Li (2008) [18]	1	1	1	1	0	1	1	1	7
Paraforou (2011) [19]	1	1	1	1	0	1	1	1	7
Stein (2011) [20]	1	1	1	1	0	1	1	1	7
Radolovich (2011) [21]	1	1	1	1	0	1	1	1	7
Aries (2012) [22]	1	1	1	1	0	1	1	1	7
Eriksson (2012) [23]	1	1	1	1	0	1	1	1	7
Hejcl (2012) [24]	1	1	1	1	0	1	1	1	7
Tsai (2013) [25]	1	1	1	1	0	1	1	1	7
Sykora (2016) [26]	1	1	1	1	0	1	1	1	7
Khalili (2016) [27]	1	1	1	1	0	1	1	1	7
Petkus (2017) [28]	1	1	1	1	0	1	1	1	7
Liu (2017) [29]	1	1	1	1	0	1	1	1	7
Adams (2017) [30]	1	1	1	1	0	1	1	1	7
Nourallah (2018) [31]	1	1	1	1	0	1	1	1	7
Chandra (2020) [32]	1	1	1	1	0	1	1	1	7
Pan (2020) [33]	1	1	1	1	0	1	1	1	7
Liljia-Cyron (2021) [34]	1	1	1	1	0	1	1	1	7
Deimantavicius (2022) [35]	1	1	1	1	0	1	1	1	7

**Table 3:** Risk of evaluating evidence based on GRADE recommendations.

Parameter	Starting Grade	Risk of Bias	Inconsistency	Indirectness	Impressision	Publication Bias	Magnitude of Effect	Dose Response	Confounding Factors	Final Grade
Survivors vs. non-survivors	2	0	0	0	0	0	0	0	0	**
Good vs. Poor outcome	2	0	−1	0	0	0	0	0	0	*
GOS 5 vs. GOS 1	2	0	0	0	0	0	0	0	0	**
GOS4–5 vs. GOS 1	2	0	0	0	0	0	0	0	0	**

**Table 4:** Summary of basic characteristics of all eligible studies. (Table 4—Abbreviations: RCC, retrtospective case controls; PCC, prospective case controls; sTBI, severe traumatic brain injury; GOS, Glasgow Outcome Scale; GOSE, Glasgow Outcome Scale Extended; CIM, Cerebral intraparenchymal monitoring; UK, United Kingdom; USA, United states of America; NR, Not Reported; CPP, Cerebral Perfusion Pressure; CPPopt, optimal Cerebral Perfussion Pressure; DC, Decompressive Craniectomy; ICP, Intracranial Pressure).

Authors	Country	SD	Patient	*n*	Intervention	Comparators	Outcome	Follow-up	NOS	Enrollment Period	Targeted CPP	Management
Yamamoto (2002) [14]	Japan	RCC	sTBI	22	CIM	Good vs. Poor outcome	GOS	NR	7	1993–2000	70 mmHg	NR
Balestreri (2006) [15]	UK	RCC	sTBI	429	CIM	Survivors vs. non-survivors	GOS	6 months	7	1992–2004	60–70 mmHg	Mixed
Ang (2006) [16]	Singapore	PCC	sTBI	40	CIM	Survivors vs. non-survivors	Mortality	6 months	7	Jan 2001–Dec 2004	NR	Mixed
Duncham (2006) [17]	USA	PCC	sTBI	18	CIM	Good vs. Poor outcome	GCS	At discharge	7	2003–2005	>60 mmHg	Mixed
Li (2008) [18]	China	RCC	sTBI	42	CIM	Good vs. Poor outcome	GOS	NR	7	2004–2007	70 mmHg	NR
Paraforou (2011) [19]	Greece	RCC	sTBI	34	CIM	Good vs. Poor outcome	GOS	6 months	7	2006–2009	70 mmHg	NR
Stein (2011) [20]	USA	RCC	sTBI	60	CIM	Survivors vs. non-survivors and Good vs. Poor outcome	GOSE	6 months	7	2005–2009	>60 mmHg	Mixed
Radolovich (2011) [21]	UK	RCC	sTBI	136	CIM	GOS 5 vs. GOS 1	GOS	6 months	7	2000–2008	NR	NR
Aries (2012) [22]	UK	RCC	sTBI	119	CIM	GOS 5 vs. GOS 1	GOS	6 months	7	2003–2009	CPP	NR
Eriksson (2012) [23]	USA	RCC	sTBI	15	CIM	Survivors vs. non-survivors	Mortality	At discharge	7	2005–2010	NR	Mixed
Hejcl (2012) [24]	Czechia	RCC	sTBI	20	CIM	Survivors vs. non-survivors	GOS	6 months	7	2005–2010	NR	Mixed
Tsai (2013) [25]	Taiwan	RCC	sTBI	40	CIM	Survivors vs. non-survivors	GOS	6 months	7	2006– 2007	>60 mmHg	Mixed
Sykora (2016) [26]	Mullticentric	RCC	sTBI	262	CIM	Survivors vs. non-survivors	GOS	6 months	7	2008–2012	NR	NR
Khalili (2016) [27]	Iran	RCC	sTBI	248	CIM	Good vs. Poor outcome	GOSE	6 months	7	2004–2007	>70 mmHg	Mixed
Petkus (2017) [28]	Lithuania	RCC	sTBI	43	CIM	Good vs. Poor outcome	GOS	6 months	7	2012–2015	CPPopt	NR
Liu (2017) [29]	UK	RCC	sTBI	195	CIM	GOS 5 vs. GOS 1	GOS	6 months	7	2004–2007	60–70 mmHg	No DC
Adams (2017) [30]	UK	RCC	sTBI	556	CIM	Survivors vs. non-survivors	GOS	6 months	7	2004–2013	NR	NR
Nourallah (2018) [31]	UK	RCC	sTBI	355	CIM	Survivors vs. non-survivors and Good vs. Poor outcome	GOS	6 months	7	2012–2016	>60 mmHg	No DC
Chandra (2020) [32]	India	RCC	sTBI	53	CIM	Good vs. Poor outcome	GOS	6 months	7	2015–2018	NR	NR
Pan (2020) [33]	China	RCC	sTBI	29	CIM	Good vs. Poor outcome	GOS	6 months	7	2014–2017	ICP threshold	NR
Liljia-Cyron (2021) [34]	UK	RCC	sTBI	200	CIM	GOS4–5 vs. GOS 1	GOS	6 months	7	2004–2007	>60 mmHg	NR
Deimantavicius (2022) [35]	Lithuania	RCC	sTBI	70	CIM	GOS4–5 vs. GOS 1	GOS	6 months	7	2017–2020	60–70 mmHg	NR

**Table 5:** Survivors vs. non-survivors Subgroup Analysis.

Country/Region	k	Mean Effect	95% Confidence Interval	τ^2^	τ
UK	3	77.52	76.18; 78.87	1.20	1.09
Singapore	1	78.80	74.84; 82.76	-	-
USA	2	73.80	72.12; 75.48	0.00	0.00
Czechia	1	84.82	83.42; 86.22	-	-
Taiwan	1	65.90	63.71; 68.09	-	-
Multicentric	1	77.70	76.79; 78.61	-	-

**Table 6:** Poor vs. Good outcome: Subgroup Analysis.

Country	k	Mean Effect	95% Confidence Interval	τ^2^	τ
Japan	1	100.10	92.13; 108.07	-	-
USA	2	76.95	69.41; 84.48	24.14	4.91
China	2	76.42	69.06; 83.77	25.40	5.04
Greece	1	78.53	67.96; 89.10	-	-
Iran	1	56.20	50.82; 61.58	-	-
Lithuania	1	86.96	82.77; 91.15	-	-
UK	1	77.60	76.60; 78.60	-	-
India	1	73.60	71.61; 75.59	-	-

## Data Availability

The dataset is available on request from authors. The raw data supporting the conclusions of this article will be made available by the authors on request.

## Supplementary Materials

The following supporting information can be downloaded at: https://www.mdpi.com/article/10.3390/brainsci15111161/s1, Table S1: PRISMA 2020 Checklist.
